# No effect of tranexamic acid on platelet function and thrombin generation (ETAPlaT) in postpartum haemorrhage: a randomised placebo-controlled trial

**DOI:** 10.12688/wellcomeopenres.14977.1

**Published:** 2019-02-05

**Authors:** Kastriot Dallaku, Haleema Shakur-Still, Danielle Beaumont, Ian Roberts, Sumaya Huque, Maria Delius, Stefan Holdenrieder, Orion Gliozheni, Ulrich Mansmann

**Affiliations:** 1 Institute for Medical Informatics, Biometry and Epidemiology, University Hospital LMU, Munich, Germany; 2 University Hospital of Obstetrics Gynaecology “Koço Gliozheni”, Tirana, Albania; 3 Clinical Trials Unit, London School of Hygiene & Tropical Medicine, London, UK; 4 Department of Obstetrics and Gynaecology, University Hospital LMU, Munich, Germany; 5 Institute of Laboratory Medicine, German Heart Center of the Technical University Munich, Munich, Germany

**Keywords:** Tranexamic Acid, Postpartum Haemorrhage, Thrombin Generation, Platelet Function

## Abstract

**Background: **Postpartum hemorrhage (PPH) is a leading cause of maternal mortality and morbidity. The WOMAN trial showed that tranexamic acid (TXA) reduces death due to bleeding in women with PPH. To determine whether TXA has pro-thrombotic effects in women with PPH, we measured endogenous thrombin potential (ETP), coagulation factors V, VIII, von Willebrand (vW), fibrinogen, D-Dimers and platelet function.

**Methods**: We conducted a sub-study within the WOMAN trial, an international randomized, parallel-group, double blind, placebo-controlled trial. Women with primary PPH were randomly allocated to receive 1 gram of tranexamic acid or matching placebo. Baseline blood samples were collected just prior to the first dose and a follow up sample was collected 30±15 minutes afterwards. We compared before and after changes in coagulation parameters between treatment groups using repeated measurement ANOVA. Change in ETP was the primary outcome. We did an intention-to-treat analysis using ANCOVA with adjustment for baseline and the time interval between the blood samples.

**Findings: **A total of 187 patients were randomized to receive TXA (n=93) or matching placebo (n=94). Six patients were excluded due to incomplete data. The reduction in ETP from baseline to follow up was 43.2 nM*min (95%CI, -16.6 to 103.1) in the TXA group and 4.6 nM*min (95%CI, -51.4 to 60.6) in the placebo group. The difference was not statistically significant (95%CI, -42.9 to 120). There were no significant effects of TXA treatment on any other parameters (ADPtest, TRAPtest, coagulation factors activity, fibrinogen levels, D-Dimer level).

**Conclusion: **We found no evidence that tranexamic acid treatment for PPH has substantial pro-coagulant effects. However, larger studies are needed to confirm or refute more modest effects.

**Trial registration:** ISRCTN76912190 (initially registered 10/12/2008, WOMAN-ETAPlat included on 28/10/2013) and NCT00872469 (initially registered 31/03/2009, WOMAN-ETAPlat included on 28/10/2013).

## Background

Postpartum haemorrhage (PPH) is a leading cause of maternal mortality world-wide and the incidence appears to be increasing ^1^. Most deaths are in low-and-middle income countries. Immediate and appropriate management of PPH is essential to reduce mortality and morbidity. The antifibrinolytic tranexamic acid (TXA) reduces bleeding by inhibiting the enzymatic breakdown of fibrin blood clots. Plasminogen is converted into the fibrinolytic enzyme plasmin by tissue plasminogen activator. TXA is a synthetic lysine analogue which blocks the lysine binding sites on plasminogen, as a result inhibits binding of plasminogen or plasmin with fibrin and thereby inhibiting fibrin degradation. It has the half-life about two hours and is excreted mostly by the kidneys ^2^. 

The CRASH 2 trial ^3^ showed that TXA significantly reduces death due to bleeding in trauma patients without any increase in thromboembolic events when given within 3 hours of injury. More recently, the WOMAN trial ^4^, showed that TXA significantly reduces death due to bleeding in women with PPH. Once again there was no evidence of any increase in thromboembolic events. On the basis of these results, TXA is recommended for the treatment of PPH and should be administered as soon as possible after onset of bleeding and within 3 hours of birth ^5^.

Although plasmin increases fibrin clot breakdown, it may also have effects on coagulation and platelets. Plasmin activates coagulation factors V and VIII ^6, 7^ and increases thrombin generation ^8^. Plasmin stimulates platelet aggregation, degranulation, complement activation and platelet activation ^6, 9, 10^. If these effects are mediated via lysine binding sites then it is possible that they might be affected by TXA administration. We conducted a sub-study within the WOMAN trial to investigate the effects of TXA on coagulation and platelets.

## Objective

To assess the effects of TXA treatment on endogenous thrombin potential (ETP) and platelet function. If plasmin activation increases ETP and stimulates platelet activation, we would expect TXA to reduce ETP and inhibit platelets.

## Methods and study design

The full ETAPlaT protocol is available from 11.

### Study design and participants

We conducted a sub-study within the WOMAN trial, an international randomized, parallel-group, double blinded, placebo-controlled trial. The study included adult women with primary PPH. The PPH diagnosis was based on the visual estimation of blood loss (>500 mL after vaginal birth or ≥1,000 mL after caesarean birth or blood loss sufficient to cause hemodynamic instability). In addition to the usual treatment for PPH, women were randomized in the study as soon as possible after informed consent had been obtained. The main criterion for eligibility was the uncertainty of clinician to use or not use TXA in a particular woman diagnosed with PPH. The study was carried out according to the guidelines of good clinical practice ^12^ and adhered to the regulatory requirements for Albania.

Ethical approvals for the study were obtained from the London School of Hygiene and Tropical Medicine (LSHTM) and the National Ethics Committee in Tirana, Albania. Brief information about the study was given to pregnant women. All eligible women underwent the informed consent procedure for the WOMAN trial as well as for the ETAPlat sub-study before randomization. The detailed consent procedure is reported at the ETAPlaT protocol ^11^. The ETAPlaT study was carried out at the Obstetric Gynaecology University Hospital “Koço Gliozheni” in Tirana, Albania. There are approximately 4500 deliveries per year in this hospital, which offers tertiary level health care and is a referral centre for other maternity hospitals at the country.

### Randomization and blinding

Women with PPH, who fulfilled the eligibility criteria and completed the consent procedures, were randomized in the study and were allocated to receive either TXA or placebo. ETAPlaT as a sub-study of WOMAN trial utilised the same randomization and blinding procedures. In summary, the trial treatment packs were identical, so both patients and healthcare workers were blinded to treatment allocation. Each box contained eight individual treatment packs, each pack contained two doses of study drugs (one dose contained: 2 vials each TXA 500mg-5 mL, or2 vials each 5mL sodium chloride 0.9%). The packs were used in sequential order by the caregiver starting from the lowest numbered pack.

### Interventions and laboratory procedures

As soon as the patient was randomized in the study, alongside with the usual treatment for PPH, the trial treatment was administered by slow intravenous injection, 1mL/minute, of 1 gram TXA or placebo. A second dose of study drugs was administered if haemorrhage did not stop after 30 minutes or restart within 24 hours of the first dose.

Baseline blood sample was collected immediately after randomization and before the first dose was administered. Three mL of venous blood was collected in hirudine (25μgr/mL) test tube - double wall (Dynabyte, Munich, Germany) for multiple electrode aggregometry (MEA) and 5 mL in tri-sodium citrate 0.106 mol/lˉ¹ (S-Monovette, Sarstedt, Germany) for coagulation tests. The same procedure for blood collection was performed at 30±15 minutes after the first dose study drug administration. Follow-up blood collection procedure was performed always before administration of the second dose of study treatment, if it was needed.

Baseline and follow-up samples were analysed for platelet function (ADPtest and TRAPtest) performing MEA with Multiplate. Details of methods used for ADPtest have been previously reported ^13^ and for TRAPtest ^14^. All material used forTRAPtest and ADPtest including Multiplate equipment, were obtained from the manufacturer (Dynabyte GmbH, Munich, Germany). The recorded platelet aggregation measured by MEA was expressed as area under curve (AUC) AU*min. The platelet function analysis using MEA was performed by the laboratory at Hospital “KoçoGliozheni” in Tirana, Albania.

The blood samples obtained in 5 mL in sodium citrate test tubes for coagulation analysis were immediately centrifuged at 3000xg for 20 min. The acquired platelet poor plasma was divided in two aliquots and preserved in deep freeze (-80 *°*C) until the laboratory analysis at the end of the study. The coagulation tests were performed at the Institute of Laboratory Medicine, German Heart Centre in Munich, Germany. The thrombin generation assay (TGA) was performed with Calibrated Automated Thrombogram (Stago Deutschland GmbH). The coagulation factors V, VIII, von Willebrand, Fibrinogen (Claus method) and D-Dimer were analyzed with SIEMENS BCS XP Coagulation Analyzer, using reagents FV and FVIII deficient plasma, Multifibren*U fibrinogen reagent, BC von Willebrand reagent and INNOVANCE ^®^ D-Dimer reagent, all reagents were obtained from Siemens Healthcare Diagnostic Products GmbH, Marburg, Germany.

### Outcomes

The primary outcome was the change (baseline versus follow-up) in ETP. Secondary outcomes included the change (baseline versus follow-up) in platelet function (ADPtest and TRAPtest) and coagulation factors V, VIII, von Willebrand, Fibrinogen, D-Dimer on baseline and follow-up blood samples. Thrombin generation was chosen as the primary outcome because it is a surrogate for coagulation activity ^15^ and factors that increase thrombin formation can potentially increase thrombotic risk ^16^.

### Statistical analysis

The statistical analysis plan to the ETAPlat Study was published and reviewed before database lock ^17^. The study evaluates the effect of TXA compared to placebo by quantifying the change over time (baseline minus follow-up) in the primary outcome ETP and a series of secondary outcomes. The study therefore compares the changes between baseline and follow up in the TXA and placebo groups (the difference in the differences).

The sample size calculation was based on the following assumptions: ETP is normally distributed with a mean of 2410 nM*min and standard deviation (SD) of 543 nM*min ^18^. We assume a decrease in ETP of 10% (241 nM*min) in the TXA group and no change in the placebo group. The calculation of the difference’s standard error is based on a correlation of 0.6 between two time points and uses the standard deviation reported by McLean ^18^. To detect an ETP difference in differences of 241 nM*min between groups at a 5% significance level with a power of 90%, two groups each with 88 patients are needed.

We compared before and after changes in coagulation parameters between treatment groups using repeated measurement ANOVA. An intention-to-treat analysis was performed by using analysis of covariance (ANCOVA) with adjustment of baseline measurement as well as adjustment of the length of time between two blood sample collection (30±15 minutes). The same analysis was carried out for secondary outcomes. Site monitoring, source data verification and trial master file review was carried out by the Sponsor and data management was performed by LSHTM using a bespoke electronic system.

## Results

**Figure 1.**
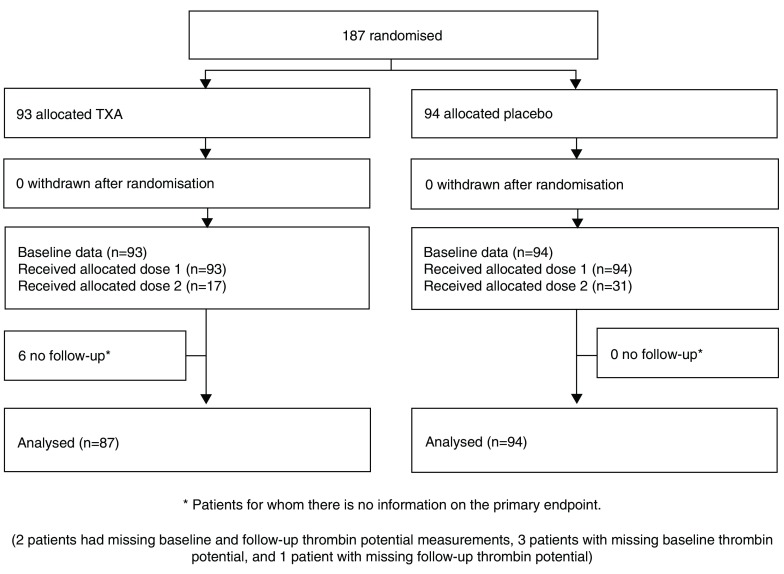
WOMAN ETAPlaT trial Consort Flowchart.

Recruitment to the ETAPlaT sub-study started on November 2013 and finished on January 2015, with final follow-up completed in March 2015. During this time 187 patients were randomized to receive TXA (n=93) or placebo (n=94). Of these 17 patients in TXA group and 31 in placebo group ( Figure 1) received a second dose of TXA or placebo. We were unable to collect baseline or follow up blood samples as emergency situation was ongoing in six patients and these patients were excluded from the analyses.

The baseline data for TXA and placebo groups were similar ( Table 1). One patient (TXA group) was known to have von Willebrand disease. The main cause of PPH in both groups was uterine atony. In the TXA group, the median (mean) time difference between both samples is 31.00 (32.98) minutes.

**Table 1.** Baseline characteristics.

	TXA group N=93	Placebo group N=94
Maternal Age - years mean (SD)	27.8 (5.6)	27.0 (5.6)
Body Mass Index- kg/m² mean (SD)	28.5 (3.1)	29.9 (4.8)
Gestational age at birth–weeks mean (SD)	38.3 (2.8)	38.2 (3.4)
FetalBirthweight – g. mean (SD)	3185 (695.2)	3222 (843.8)
Hemoglobin - g/dL ^+^ mean (SD)	10.8 (1.8)	11.4 (1.6)
Fibrinogen - g/L mean (SD)	3.6 (1.1)	3.5 (1.2)
Platelet count -10³/mm³ mean (SD)	229.3 (80.4)	233.7 (80.1)
Blood lost mL mean (SD)	863.2 (270.7)	893.1 (219.9)
Vaginal births Labor stages - First stage(hrs) Mean (SD) [N] Second stage (min) Third stage (min)	6.7 (4.3) [71] * 40.5 (26.0) [67] * 10.9 (9.1) [66] *	7.8 (4.1) [68] * 48.5 (31.6) [66] * 13.5 (12.4) [65] *
Parity : N (%) Nullipara Multipara	57 (61.3 %) 36 (38.7 %)	60 (63.8 %) 34 (36.2 %)
Type of delivery: N (%) Vaginal Caesarean	65 (69.9%) 28 (30.1%)	65 (69.2%) 29 (30.8%)
Primary cause PPH: N (%) Placenta previa Surgical trauma /tears Uterine atony Other Unknown	11 (11.8%) 10 (10.8%) 71 (76.3%) 1 (1.1%) 0 (0%)	14 (14.9%) 19 (20.2%) 59 (62.8%) 1 (1.1%) 1 (1.0%)
Preeclampsia: N (%) Yes No	11 (11.8 %) 82 (88.2 %)	7 (7.4 %) 87 (92.6 %)
Chorioamnionitis: N (%) Yes No	5 (5.4 %) 88 (94.6 %)	6 (6.4 %) 88 (93.6 %)
Placental abruption: N (%) Yes No	9 (9.7 %) 84 (90.3 %)	5 (5.3 %) 89 (94.7 %)
Anaemia: N(%) Yes No	34 (36.6 %) 59 (63.4 %)	23 (24.5 %) 71 (75.5 %)
Previous PPH: N (%) Yes No	3 (3.2 %) 90 (96.8 %)	6 (6.4 %) 88 (93.6 %)
Hematologic disease N (%) Yes No	1 (1.1 %) 92 (98.9 %)	0 (0%) 94 (100 %)
Treatment with antithrombotics N (%) Yes No	0 (0%) 93 (100 %)	2 (2.1 %) 92 (97.9 %)

* Patients vaginal delivery; ^+^ p-value Wilcoxon-Mann-Whitney-Test: 0.01

In the placebo group, the median (mean) time difference between both samples is 30.00 (31.74) minutes. In TXA group, the minimum (maximum) time difference between both samples is 20.00 (45.00) minutes. In placebo group, the minimum (maximum) time difference between both samples is 15.00 (63.00) minutes. There is no evidence that time differences differ between both treatment groups (Wilcoxon-Mann-Whitney-Test, p = 0.1336).

### Primary outcome - results

The change in ETP (expressed in nM*min) between baseline and follow-up was 43.2 (95%CI, -16.6 to 103.1) in the TXA treated group and 4.6 (95%CI, -51.4 to 60.6) in the placebo group. The difference in differences (DiD) of 36.63 (TXA minus Placebo) was not statistically significant (p _raw_ =0.350, 95%CI _raw_, -42.9 to 120.0). The detailed values are given in Table 2.

**Table 2.** Effect of TXA on primary and secondary endpoints.

	Baseline Mean (SD)	Follow-Up Mean (SD)	Baseline/Follow-Up Mean Difference (95% CI)	DID: TXA / Placebo groups Mean DID ( 95%CI) p Value
**ETP**(nM*min)				
TXA (N=87)	1537 (375.9)	1494 (369.1)	43.2 [-16.6; 103.1]	36.63 [-42.9; 120.0] p _raw_ = 0.350 * 29.81 [-47.8; 107.4] p _adj_ = 0.453 **
Placebo (N=94)	1491 (378.7)	1487 (390.5)	4.6 [-51.4; 60.6]	
**ADPtest**(AU*min)				
TXA (N=89)	1043.0 (343.6)	964.7 (312.4)	78.0 [15.4; 140.6]	13.2 ( -65.8; 92.2) p _raw_= 0.7 * -0.9 ( -72.7; 70.9) p _adj_ = 1.0 **
Placebo (N=91)	961.6 (339.6)	896.8 (356.8)	64.8 (15.8; 113.7)	
**TRAPtest**(AU*min)				
TXA (N=89)	1199 (362.3)	1093 (300.3)	106.2 (38.5; 174.0)	20.9 ( -65.3; 107.1) p _raw_= 0.6 * 1.5 ( -72.8; 75.7) p _adj_ = 1.0 **
Placebo (N=91)	1156 (347.9)	1070 (336.8)	85.3 (31.2; 139.4)	
**Factor V (%)**				
TXA (N=88)	103.4 (27.7)	103.4 (25.7)	0.1 (-4.6; 4.8)	-4.2 ( -10.0; 1.7) p _raw_= 0.2 * -4.9 ( -10.4; 0.7) p _adj_ = 0.1 **
Placebo (N=94)	100.2 (28.5)	95.9 (29.2)	4.3 (0.7; 7.8)	
**Factor VIII (%)**				
TXA (N=88)	221.9 (102.4)	216.1 (87.4)	5.9 (-9.5; 21.2)	5.9 ( -13.9; 25.6) p _raw_= 0.6 * -0.9 ( -18.7; 17.6) p _adj_ = 1.0 **
Placebo (N=94)	195.2 (87.0)	195.2 (87.4)	0.0 (-12.7; 12.7)	
**Factor vW (%)**				
TXA (N=88)	219.4 (89.8)	222.2 (89.1)	-2.8 (-15.2; 9.7)	-0.1 ( -16.9; 16.7) p _raw_= 1.0 * 1.3 ( -15.0; 17.5) p _adj_ = 0.9 **
Placebo (N=94)	212.6 (92.6)	215.3 (92.0)	-2.7 (-14.1; 8.8)	
**Fibrinogen (g/L)**				
TXA (N=87)	3.64 (1.09)	3.59 (1.07)	0.05 (-0.1; 0.2)	-0.08 (-0.29; 0.12) P _raw_= 0.4 * -0.09 (-0.28; 0.1) p _adj_ = 0.4 **
Placebo (N=93)	3.49 (1.2)	3.36 (1.1)	0.13 (-0.01; 0.27)	
**D-Dimer (mg/L)**				
TXA (N=88)	7.4 (9.3)	7.8 (10.2)	-0.4 (-1.1; 0.3)	0.9 ( -1.3; 3.0) p _raw_= 0.4 * 1.5 ( -0.2; 3.1) p _adj_ = 0.1 **
Placebo (N=94)	9.6 (24.6)	10.8 (17.7)	-1.3 (-3.4; 0.8)	

Population: Patients were difference could be calculated (N); DID: Difference in Differences Analysis: * raw (simple 95% CI of DID); ** adjusted for baseline and time between samples

### Secondary outcomes - results

Table 2 also summarizes the exploratory results for the secondary outcomes. The change in platelet activity (expressed in AU*min) in the ADPtest was larger with TXA (mean change 78.0, 95%CI, 15.4 to 140.6) compared to placebo group (mean change 64.8, 95%CI, 15.8 to 113.7), but with no significant difference in difference (DiD 13.2, 95%CI, -65.8 to 92.2). The mean difference of the TRAPtest for the TXA group was 106.2, (95%CI, 38.5 to 174.0) compared to the placebo group 85.3, (95%CI, 31.2 to 139.4). The difference in difference was not significant (DiD -20.9, 95%CI, - 65.3 to 107.1). There was no significant DiD between treatment groups for coagulation factors activity (expressed in % of the norm). The results are as follows: factors V DiD -4.2, 95%CI, -10.0 to 1.7, factor VIII DiD 5.9, 95%CI, -13.9 to 25.6, and von Willebrand factor DiD -0.1, 95%CI, -16.9 to; 16.7. No significant difference in difference was observed for Fibrinogen, expressed in g/L, (DiD -0.08 with 95%CI, -0.29 to 0.12) or D-Dimer, expressed in mg/L, (DiD 0.9 with 95%CI, -1.3 to 3.0). Detailed changes in the single treatment groups are presented in Table 2.

## Discussion

We found no evidence that tranexamic acid (TXA) has large effects on thrombin generation or platelet function. However, we cannot exclude the possibility of more modest effects. Thrombin plays a crucial role in coagulation ^15^ and increased thrombin generation is associated with an increased risk of thrombosis ^19^. Plasmin has been shown to increase thrombin formation in the blood of healthy volunteers *in vitro* ^8^. An increase in thrombin generation by plasmin was also reported during treatment with tissue plasminogen activators ^20^. ETP was decreased about 30% after administration of very effective anticoagulant agents such as low-molecular-weight heparin, in postpartum period soon after caesarean delivery ^21^ and also during pregnancy at an *in-vitro* study ^22^. In our study there was a small decrease (3%) in ETP with TXA administration (DiD 36.63, 95% CI, -120; 42.8) that was not statistically different to that seen in the placebo group. This study provides no evidence TXA has a pro-thrombotic effect.

Plasmin has multifactorial pro-coagulant effects on platelet activation ^10, 23^. Activation of platelets may contribute to thrombus formation ^24^. The evaluation of platelet activity using MEA with ADPtest and TRAPtest provides information about the thrombotic risk. The reported range for healthy volunteers of ADPtest was 483 to 1173 AU*min. The reported range for the TRAPtest was 897 to 1469 AU*min. ^25^. The observations of our study are comparable within both treatment groups and in a good fit with the results of Rubak ^25^. They show a modest decrease in platelet activity in both tests in TXA group compared to placebo, but the difference was not statistically significant. Once again, these results provide no evidence that TXA has pro-thrombotic effects.

In the last trimester of pregnancy, plasma levels of plasminogen and fibrinogen increase by about 50% whilst levels of plasminogen activator inhibitors 1 and 2 increase 3-fold and 25-fold, respectively ^7^ . Immediately following delivery there is early fibrinolytic activation and this can be inhibited by TXA ^26^. The inhibition of fibrinolysis with TXA has the potential to increase thrombotic risk ^27^. By reducing fibrinolysis, TXA can help to maintain fibrinogen levels. In our study, the drop in fibrinogen (DiD -0.08, 95%CI -0.29; to 0.12) was smaller in the TXA group but again the difference was not statistically significant.

### Study limitations

The study was designed to prove a difference (more relevant changes with the use of TXA compared to placebo) and was not planned to establish therapeutic equivalence. There are no predefined therapeutic equivalence bounds which would allow an objective comparison between derived DiD confidence intervals. The study uses a large series of secondary endpoints and multiple testing performed in an explorative setting. In the ETAPlaT study, although we did not measured plasmin directly but evaluated thrombin generation and platelet function as an indirect effect of TXA on plasmin inhibition. Some post-randomization exclusions were performed, because of the emergency situation of PPH it was difficult to collect the baseline or follow up or both blood samples.

## Conclusion

Although the inhibition of fibrinolysis with TXA has the potential to increase thrombotic risk, we found no increase in thrombin generation and no increase in platelet activity with TXA.

### Ethics approval and consent to participate

Ethical approval for WOMAN ETAPlaT protocol was obtained from the London School of Hygiene and Tropical Medicine Ethics Committee, United Kingdom, and by the National Ethics Committee in Tirana, Albania. ETAPlaT study was undertaken according to local regulatory requirements, and adhered the ICH-GCP guidelines. The consent procedure was approved by each Ethics Committee and is detailed in the previously published WOMAN trial and ETAPLaT protocols ^4, 11^. Briefly, consent was obtained from a woman if her physical and mental capacity allowed (as judged by the treating clinician). If a woman was unable to give consent, proxy consent was obtained from a relative or representative (who was not involved in the trial and was approved by the hospital). If a proxy was unavailable, then as permitted by local ethics approval, consent was deferred. When consent was deferred or given by a proxy, the woman was informed about the trial as soon as possible, and consent was obtained for ongoing data collection, if needed.

## Data availability

The anonymised data used for this publication is available from the freeBIRD data portal at https://freebird.lshtm.ac.uk/index.php/data-sharing/downloads/etaplat/ following free registration: http://www.doi.org/10.17037/DATA.00000970 ^28^. Data are available under an Open Data Commons Attribution License (ODC-By) licence.

## Reporting guidelines

This study is compliant with CONSORT guideline recommendations ^29^.
