# Supplementary material for: No effect of tranexamic acid on platelet function and thrombin generation (ETAPlaT) in postpartum haemorrhage: a randomised placebo-controlled trial
Source: Wellcome Open Res. 2019 Feb 5;4:21. [Version 1] doi: 10.12688/wellcomeopenres.14977.1 (PMC6556984; doi:10.12688/wellcomeopenres.14977.1)
Supplement: Peer review history

# Peer review: Reviewer response for version 1

**DOI:** 10.21956/wellcomeopenres.16336.r35666

**Published:** 2019-06-04

**Authors:** Homa K. Ahmadzia

**Affiliations:** 1 Division of Maternal-Fetal Medicine, Department of Obstetrics and Gynecology, School of Medicine and Health Sciences, George Washington University, Washington, DC, USA

**Summary**:

 This article by Dallaku *et al*. is a subgroup analysis of the WOMAN trial, in which women with diagnosed PPH were randomly assigned to receive placebo or TXA (1g IV) for treatment. The objective was to assess effects of TXA on thrombin generation and platelet function, testing the hypothesis that TXA would reduce endogenous thrombin potential (ETP) and inhibit platelets.

 'Baseline' blood samples were drawn prior to initial drug administration and 'follow up' samples approximately 30 min after first drug administration. A majority of patients received 1g TXA but a third or less received a second 1g due to continued bleeding.

 The results showed that there was a slight decrease in ETP, but certainly no increase after TXA administration *in vivo*. In addition, in the TXA group comparing post drug to pre drug levels there was no increase in platelet activation or other coagulation markers/assays (d-dimer, factors V/VIII/vW and fibrinogen).

**General Comment:**

 Overall important contribution to the literature since it adds new information about drug pharmacodynamics in the setting of treatment for PPH, and supports that TXA does not increase hypercoaguable profile in the peripartum period.

**Suggestions for improvement:**

 Although this was a novel approach to use thrombin generation assay (TGA) in the peripartum period, the results focused solely on ETP. The conclusion would be more robust if similar findings were true for other parameters on TGA (i.e. include analysis on comparisons of lag time and time to peak pre/post drug, perhaps as supplement if not main table/figure).

 Also a figure (with individual data points) to show distribution of the ETP values in pre/post drug groups would be helpful as well to illustrate the data.

 I am not sure the 'difference in difference' statistical approach is the clearest way to represent the analysis, especially when the post minus pre drug levels were what was focused on as the key findings.

 I agree with Reviewer #1 that a separate analysis of the women who received 2 grams would be of interest to see if any difference was seen from post drug dose #1 to post drug dose #2 and the difference in baseline from the dose #2. That would make the data more robust if there was no difference seen in that ETP as well, even if not powered to look at that as primary outcome.

**Consideration for limitation/discussion:**

 I also found the estimated blood loss to be on lower end for PPH, possibly due to the fact that more than 2/3 of patients in both groups were vaginal deliveries. I would include in the limitations that this is a selective group of PPH and some groups may not define as such with more recent definitions ( US ACOG Revitalize which is >=1000 mL regardless of mode of delivery). However, this could also be seen as a strength to the study given if these findings could support preliminary safety data using TXA for 'mild' PPH cases, which is clinically more useful to intervene rather than severe PPH when other coagulopathies can result simply from high volume blood loss and when in lower resource settings blood transfusion is not readily available. 

**Minor comments:**

- Table 2 - ETP DID TXA should be 38.6 and not 36.6.
- Page 7 - typo to remove 'd' in 'study limitations:...'we did not measure[d] plasmin directly'. 

I have read this submission. I believe that I have an appropriate level of expertise to confirm that it is of an acceptable scientific standard.

# Peer review: Reviewer response for version 1

**DOI:** 10.21956/wellcomeopenres.16336.r34770

**Published:** 2019-02-15

**Authors:** Anne-Sophie Ducloy-Bouthors, Tournoys Antoine

**Affiliations:** 1 French Society of Anesthesia and Resuscitation (SFAR), Paris, France; 2 Biology Hemostasis Pathology Center, Lille University Hospital, Lille, France

We thank the authors for the quality of their trial and scientific data, the obstetric and anesthesia and intensive care community may benefit from this article.

 The authors analyzed the effects of tranexamic acid (TXA) administration on endogenous thrombin potential (ETP) and other coagulation factors and markers compared to placebo.

 The study is a WOMAN Trial sub study.  The tubes were sampled out of the patients included in this international randomized double blind trial. One Albanian center was the site where samples were taken and biological products were centrifuged and frozen. Biological results were obtained by laboratory tests in a German lab in Munich.

 The authors showed no impact of TXA administration compared to placebo on ETP and platelet function.  According to the authors, the stability of endogenous thrombin potential  and platelet activation tests suggests that TXA has no pro-thrombotic effect. WOMAN trial clinical and ETAPlat biological data support the safety of TXA use in obstetric hemorrhage.

 My major comment is that the selected population is a mild haemorrhage population developing minor or no coagulopathy  as suggested by: 

- minor blood loss baseline in both groups : 863.2 (270.7) and 893.1 (219.9)mL;
- baseline plasma fibrinogen level above 3g /L predicting a moderate thrombin generation and moderate final severity and 
- moderate decrease in baseline to follow-up fibrinogen plasma level and platelet count decrease;
- minor fibrinolysis as showed by the moderate decrease of baseline factor V and moderate increase of baseline D Dimers.

Thus, to consider this population data, it would be of interest to clarify the conditions of substudy recruitment regarding in one part the center’s recruitment and on the other part WOMAN trial’s global population:

- Was the series of sampled patients a consecutive series of all patients included during the substudy period?
-  The substudy period was shorter than the study period. Did the center continue its recruitment to complete clinical part of the study or did the center stop after the time when substudy recruitment was completed?

It would be of interest to describe the final volume of PPH in order to give information about the proportion of severe patients. 

 My second comment concerns the mild ETP level reached in the study:

Thrombin generation analysis was the primary criteria.

 The pre-supposed normal value of thrombin generation that was used to calculate the sample size of the sub study was 2410 +/- 543 nM*min”. Joly et al had observed a similar reference value during the third trimester of pregnancy 2123 ± 335 (nM.min) (CAT Thermoscientific Labsystems  assay) ^1^ as well as Macey et al in their pregnant control at 38 Weeks of pregnancy 2515 [2289 – 2726]nMIIa (CAT STAGO assay) ^2^.  

 It is important to clarify if these reference values had been given by the reagent’s supplier or tested by the involved laboratory and if the population recruited to measure these reference values were both gender or only women of child-bearing age or only normal pregnant women or normal immediate postpartum women.

  

- Both baseline and follow-up ETP observed in the ETAPlat substudy groups were lower than the reference value, see here.
- There was no significant decrease from ETP baseline to follow-up in both groups. Follow up was sampled 30 minutes after baseline: maybe too early to observe the changes in thrombin generation as the maximal PPH-induced coagulation changes were observed 2 hours after the beginning of PPH ^3^.
- Regarding the therapeutic effect of TXA focused on plasmin generation decrease, the authors based their hypothesis on the indirect impact of thrombin potential reduction due to plasmin generation reduction. It had been previously observed that TXA inhibits D-Dimers increase with no effect on fibrinogen and factor II decreases (substrate and proenzyme of thrombin)(3).  As suggested by the TRACES trial pilot study ^4^, if this mechanism exists, it would be better to select coagulopathic patients and higher doses of TXA.  Following this suggestion, it could be of interest to analyze the thrombin generation and fibrinogen and platelets data from the 18 % and 33 % of patients receiving 2 allocated doses. Although the number of these patients is limited, the second dose’s administration shows that PPH is ongoing and thus concerns the more severe ones with higher thrombin and plasmin generation and TXA dose effect than the 1g population. Pharmacobiological TRACES trial is aimed to provide more detailed data in this setting ^4^.

Finally platelet activation assays were potentially useful to better understand the evolution of primary hemostasis in PPH.

Conclusion : regarding my comments concerning the selected population and the TA dose, I would ask the authors to change their title and conclusion by introducing the two important details:  “ 1g TXA” and “ mild postpartum haemorrhage”.

We have read this submission. We believe that we have an appropriate level of expertise to confirm that it is of an acceptable scientific standard.

## References

[1] MaceyMGBevanSAlamSVergheseLAgrawalSBeskiSThuraisinghamRMacCallumPK: Platelet activation and endogenous thrombin potential in pre-eclampsia. *Thromb Res*.2010;125(3) : 10.1016/j.thromres.2009.09.013 e76-81 10.1016/j.thromres.2009.09.013 19822350

[2] JolyBBarbayVBorgJYLe Cam-DuchezV: Comparison of markers of coagulation activation and thrombin generation test in uncomplicated pregnancies. *Thromb Res*.2013;132(3) : 10.1016/j.thromres.2013.07.022 386-91 10.1016/j.thromres.2013.07.022 23962423

[3] Ducloy-BouthorsASDuhamelAKipnisETournoysAPrado-DupontAElkalioubieAJeanpierreEDebizeGPeynaud-DebayleEDeProstDHuissoudCRauchASusenS: Postpartum haemorrhage related early increase in D-dimers is inhibited by tranexamic acid: haemostasis parameters of a randomized controlled open labelled trial. *Br J Anaesth*.2016;116(5) : 10.1093/bja/aew021 641-8 10.1093/bja/aew021 27106967

[4] Ducloy-BouthorsASJeanpierreESaidiIBaptisteASSimonELannoyDDuhamelAAllorgeDSusenSHennartB: TRAnexamic acid in hemorrhagic CESarean section (TRACES) randomized placebo controlled dose-ranging pharmacobiological ancillary trial: study protocol for a randomized controlled trial. *Trials*.2018;19(1) : 10.1186/s13063-017-2421-6 149 10.1186/s13063-017-2421-6 29490690PMC5831219
